# A Novel Tactile Learning Assistive Tool for the Visually and Hearing Impaired with 3D-CNN and Bidirectional LSTM Leveraging Morse Code Technology

**DOI:** 10.3390/bioengineering12030253

**Published:** 2025-03-03

**Authors:** Shabana Ziyad Puthu Vedu, May Altulyan, Pradeep Kumar Singh

**Affiliations:** 1 Department of Computer Science, College of Computer Engineering and Sciences, Prince Sattam Bin Abdulaziz University, Al Kharj 11942, Saudi Arabia; 2 Department of Computer Engineering, College of Computer Engineering and Sciences, Prince Sattam Bin Abdulaziz University, Al Kharj 11942, Saudi Arabia; m.altulayan@psau.edu.sa; 3 Department of Computer Science and Engineering, Central University of Jammu, Jammu and Kashmir 181 143, India; pradeep.cse@cujammu.ac.in

**Keywords:** learning assistive tool, human–computer interaction, hearing and visually impaired, tactile learning, 3D convolutional neural network, LSTM, word error rate, morse code

## Abstract

Educating deafblind children is a highly specialized field that requires computer-assisted learning tools to address the challenges of auditory and visual impairments. The objective is to reduce their difficulties in communication with their peers and to empower them to learn independently in a classroom environment. Braille and assistive tools have become profoundly beneficial for deafblind children, serving as an essential means of communication and knowledge acquisition, enabling them to live independently. This study aims to develop an assistive tool that bridges the limitations of conventional tactile methodologies by incorporating the latest artificial intelligence techniques, enabling children to learn with greater ease. The research leverages Morse code technology to facilitate communication with deafblind children. The speaker’s lip movements are converted into text using the deep learning techniques of a 3D convolutional neural network and a bidirectional long short-term memory neural network. Experimental evaluations of this text conversion model show a word error rate of 2% and an accuracy rate of 98%. The text is then converted into Morse code and communicated to the deafblind child through a wearable device. The significance of this assistive tool lies in its discreet design, resembling a smartwatch. Adolescents can wear the proposed wearable device confidently without feeling self-conscious or embarrassed.

## 1. Introduction

Disability in children is a significant concern for parents everywhere around the world. Life tends to be a challenge for children who are visually impaired and require assistance even to perform day-to-day activities. According to statistics for the year 2020, there are 1.5 million blind children worldwide, and around 500,000 children become blind every year [1]. Deaf blindness is “Concomitant hearing and visual impairments, the combination of which causes severe communication issues and other developmental and educational needs that cannot be accommodated in special education programs designed solely for children who are either blind or deaf” [2]. Approximately 0.2–2% of the world’s population suffers from deaf blindness [3]. Hearing and visually impaired children have poor expressive and receptive speech. Such children with a severe lack of auditory input develop a lack of communication skills that severely impair their education [4]. Their means of communication with family, friends, and the outside world are cut off due to their disability. Education for disabled children, physically or mentally, is a great challenge faced by educational systems all around the world. The challenges faced by disabled children in communicating with their peers and classmates are beyond all limits. The teachers introduce new words to deafblind children with the physical touch of the objects. Visually impaired children learn braille, a tactile reading system that enables blind people read. Braille is a tactile-based learning tool that has raised dots to represent letters for blind people to touch and read, which makes it easier for them to classify letters [5]. The blind children understand the models of real-world objects by the tactile method. The children who are auditorily and visually impaired find it difficult to understand the explanations given during the tactile teaching method. Teaching abstract concepts in mathematics and science is challenging for deafblind children. Versatile assistive tools are available to aid in visual, auditory, communication, mobility, and cognitive impairments [6]. Computer-aided assistive tools based on object detection, text detection, and audio converters have enabled blind people to communicate and commute [7] easily. Tactile displays such as braille, e-readers, and math tools like talking calculators are a few to add to the list of assistive technology-based learning tools specially developed for blind people to improve the quality of learning [8]. Sensory augmentation provides additional information to a human’s sensory channel. The haptic devices focus on tactile communication and transmit information through touch [9]. Education for deafblind children is highly challenging compared to blind children. Tactile modeling strategies educate blind children. The hearing and visually impaired need a special tactile teaching methodology to identify objects and words using their sense of touch at an early age. In tactile modeling, the teacher introduces the words using the sense of touch. This method teaches children objects and body parts at an early age. Though tactile sign language is more effective for deafblind students, some abstract concepts taught at higher school levels are challenging to teach disabled children through tactile sign language. This research study uses artificial intelligence concepts and wearable devices to empower visually and auditorily impaired students to follow the tactile teaching methodology effortlessly. Overwhelming tactile stimuli and misplaced braille labels were some issues raised by students in a research study conducted in 2022 [10]. The proposed assistive tool lipreads the conversation and converts it to the Morse code generated by the wearable device worn by people with disabilities. A wearable device that can transmit Morse code enables the children to understand the speech given in the classroom and help them follow the classes better. This research study aims to develop a tool to interpret speech into Morse code for hearing and visually impaired children. This research study proposes to develop an integrated automated lip-recognition system with a wearable device. A novel methodology for visually and hearing-impaired students to follow the lectures in the classroom, minimizing the tactile strategy, is proposed in this research study. The Kingdom of Saudi Arabia has introduced institutions such as Al-Noor and Al Amal. In 1962, The Directorate of Education established the Al-Noor Institute of the Blind in Makkah to provide education, training, care, and recreational services to children with vision impairments [11]. Al-Amal Institute for the deaf and mute has institutes all over Saudi Arabia to educate deaf and mute students. According to statistics from a study published in 2016, Al Nour has ten institutes and 625 students, whereas Al Amal has 23 institutions with 2855 students [12]. The Ministry of Education in Saudi Arabia shows keen interest in assisting speech, auditory, and visually impaired students in receiving quality education at par with non-disabled students.

## 2. Literature Review

Recent technological advances in artificial intelligence have made room for several valuable contributions to overcome communication hurdles for people with disabilities. Assistive tools, such as wearable devices, are developed for individuals to overcome their disability [13]. The proposed tool to convert and interpret the speech as Morse code to the disabled children is a breakthrough in the traditional tactile teaching method worldwide, including in Saudi Arabia. In recent years, artificial intelligence-driven healthcare models have found applications in different fields of medicine. Be it preventive medicine, patient remote monitoring systems, diagnostic tools, or healthcare informatics, artificial intelligence technology has always played a significant role [14]. As robust algorithms have replaced traditional machine learning algorithms with improved efficiency in almost every healthcare domain, lip recognition was no exception. Automated lip-reading systems are generally developed based on the convolution neural network and recurrent neural network (RNN) models. LipNet is a sentence-level lip reading model developed with RNN and temporal classification loss trained end to end. The study maps videos of varying durations to text by spatiotemporal convolutions, recurrent networks, and temporal classification loss. With the GRID Corpus dataset and the gated recurrent unit, the model achieves 95.2% accuracy in sentence-level, overlapped speaker split tasks [15]. The extraction of the lip features from the image frame using VGG and then recognizing the words exhibits an accuracy of 88.2% in the test dataset, which is 3.3% higher than the general CNN-RNN model [16]. The study proposes hybrid neural network architecture with CNN and bidirectional LSTM (BiLSTM) for lip reading. The eight-layer CNN layer extracts the features of the raw mouth regions from the isolated video clips. The BiLSTM discovers the patterns in the two directions from the frames and the Softmax function to predict the recognition result [17]. Speech recognition using visual features is challenging as speakers vary in accent, facial expressions, and positions. This challenge can be overcome by extracting lip movement along with Mel Frequency Cepstrum (MFCC) features for audio to predict the occurrence of the words. Multi-layer perceptron algorithm gives an accuracy of 91% [18]. Lip reading networks are computationally expensive to build and require ample storage for data. Therefore, GhostNet, a lightweight network, uses a local cross-channel interaction strategy for the dimensionality reduction in features extracted from lip movements. The temporal features of the lip sequences are an input to the GRU network. The study aimed to prove that an improved Efficient-GhostNet and GRU model reduces the parameters in the neural network in a real-time environment [19]. Human speech perception is a multi-modal process, where improving the efficiency of automatic speech recognition systems was a challenge until the advent of audio-visual tools and neural network architectures. In the early years, machine learning algorithms for feature extraction combined with Hidden Markov Models (HMMs) could concisely convey context information to signals [20]. The proposed research study aims to develop a novel methodology with a customized stacked bidirectional LSTM model for lip reading with a GRID Corpus dataset along with the hardware design to convert the read text to Morse code.

Students who are visually and auditorily impaired frequently encounter barriers in a classroom environment. Visually and auditorily impaired children are deprived of the opportunity to gain knowledge and skills through traditional training methods available to their regularly abled peers. Assistive technology is a general term that refers to all products and services that assist disabled students [21]. This assistive technology is a boon to visual and deaf students who find tactile learning methods challenging. AT can be categorized based on the device’s features, design complexity, and cost. As far as visually impaired students are concerned, providing them with theoretical content and training them for a work environment is challenging. Refreshable Braille displays, Braille readers and note-takers, Braille personal digital assistants, OCR software and hardware, talking calculators, and specialized e-readers are some assistive learning tools for visually impaired students [22].

AT tools like talking calculators and specialized e-readers that convert the e-books to audio format are futile for an auditorily and visually impaired student. The products that give output in braille format are helpful for the hearing and visual impairment of the student. Visually and hearing-impaired students are familiar with assistive technology such as braille readers, braille slate, tactile screens, and note-takers [23]. Hence, assistive technology is successful when the model has a good design, development, implementation, and testing plan. Such tools benefit physically impaired students only with adequate monitoring. Training sessions for the students are necessary to ensure the effective use of the tool and reap its full benefit. Stigma is a fact that should be considered while developing an assistive tool. The students should not be embarrassed to use a tool as adolescents [24]. Considering such sensitive issues, this proposed system aims to develop a wearable device that connects to the speech decoder device, converting the speech to Morse code.

## 3. Architecture of Learning Assistive Tool

**Figure 1:**
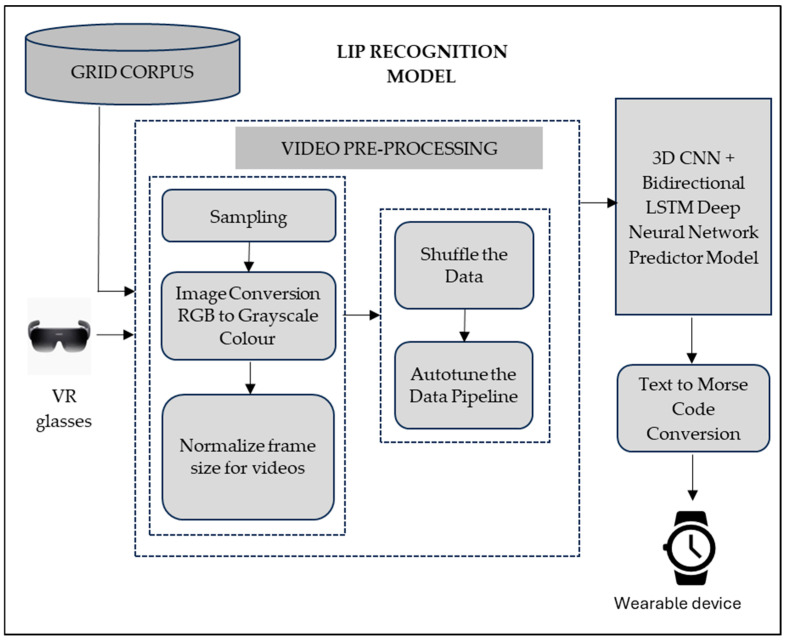
Block diagram of the proposed learning assistive tool for the visual and hearing impaired.

The proposed speech decoding system as a learning assistive tool has three modules. Video preprocessing is carried out by the first module of the proposed system. The subsequent module performs the automated recognition of lip movements with the deep neural network that predicts the words from the lip movement. The last module implements the Morse code generation corresponding to the spoken words. Figure 1 showcases the block diagram of the proposed model of learning assistive tool for the deaf-blind children. Automated lip-reading technology comprises human–computer interaction and virtual reality technology. A VR environment captures the lip movements of the speaker and, in turn, analyses the speaker’s emotions or state of mind. Visual speech recognition techniques enhance virtual reality concepts. Visual speech recognition converts lip movements into readable text [25]. Deep learning models have a significant role to play in speech recognition tools. This proposed speech decoder system builds robust deep-learning models that result in high predictive performance. Experimental results prove that the deep learning models of 3D CNN and bidirectional LSTM perform lip reading classification at a high accuracy rate. The Morse code converter module of the speech decoder system converts text identified from speech into Morse code. The wearable device communicates the Morse code to people with disabilities. The proposed tool transcends the communication barriers for hearing and visually impaired children. The proposed system significantly enhances the child’s ability to follow tactile learning. Figure 1 shows the block diagram of the proposed assistive tool for the visual and hearing impaired.

### 3.1. Dataset

The dataset used for this research work is the GRID Corpus [26]. GRID, a multitasker audio-visual sentence corpus, is an open-source dataset frequently used by researchers for speech perception. The dataset consists of high-quality audio and video (facial) recordings of 1000 sentences spoken by 34 speakers. Amongst the speakers, 18 are male and 16 are female. Video files are available in two formats. One with usual quality (360 × 288; ~1 kbit/s) and the other with high quality (720 × 576; ~6 kbit/s). We have utilized 40 high-quality videos from the GRID Corpus dataset to train and test the model. The number of frames used as an input to the model is 75 frames per video. The dataset has 100 ASCII characters.

### 3.2. Data Pre-Processing

The videos comprise color images in RGB channels with varying numbers of frames. The videos were segregated into frames for mapping to the lip alignments. The video frames are converted to grayscale and cropped with a window size of 190:236 and 80:220. All the frames in the pre-processing videos are 140 pixels × 40 pixels. The number of frames per video is normalized by computing the mean value and the Standard deviation. Data are pre-processed by shuffling and autotuning the images. Data shuffling is an inevitable task while training sequential data, failing which the model learns spurious patterns based on the sequential order of words occurring in the data. There is a risk of model overfitting when the model learns the local patterns without generalizing and learning the entire train data. Shuffling ensures that the model trains on the data in a random order, preventing it from learning any relationships from the data. Randomizing the order of the data allows the model to generalize better across different samples.

**Figure 2:**
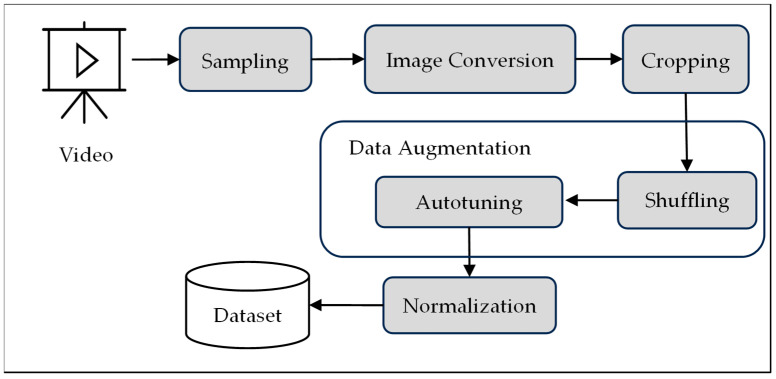
Block diagram of video pre-processing module of proposed speech decoder system.

The model is less likely to overfit test data when trained on versatile samples in each epoch. Shuffling a batch of 500 videos ensures that the model learns across a broader range of samples encountered during each batch, improving the ability to generalize the model. In large datasets, there is a high risk of bottlenecks in the data pipeline while fed as input to the model. The risks of bottlenecks in the data pipeline are eliminated by overlapping data processing techniques carried out by multi-threading and parallel processing. Input given to the model are batches of data. While the model is processing the current batch, the next batch is being prepared in the background and is ready to be loaded. Overlapping of data processing prevents the processor from remaining idle and improves resource utilization during the training. This overlapping of processes considerably reduces the computational costs. The prefetch buffer size is automatically tuned during runtime to optimize the data loading and training time. In a nutshell, the autotune prefetches the batches of data in the background while training the model to improve the GPU’s processing speed. Figure 2 exhibits the block diagram of the video pre-processing module of the speech decoder system.

### 3.3. Speech Decoder Model

The lip-reading dataset is a collection of speech videos labeled with text subtitles, which is the interpretation of the speech in the corresponding video. The speech decoder modules predict the words uttered by the speaker with the deep learning model. The words spoken by the speaker are converted into a text format by the deep learning model. The movement of the lips is mapped to the lip alignment data corresponding to each word uttered. The dataset includes mapping the alignment of the words with 3D CNN and bidirectional LSTM to train the model. The model then predicts the new sample videos from the test data with the knowledge gained from the dataset. The data pre-processing by shuffling overcomes the issue of learning spurious patterns from the train data. The 3D CNN model extracts the features of the lip movement and feeds it as an input to the bidirectional LSTM for word prediction in the sentence.

The decoder architecture shows three blocks, each having a Conv3D layer, rectilinear activation function, and Max pooling layer. The Conv3D layer extracts the features from the images using filters with a kernel of size three and padding all sides with zero to make the output dimension the same as the input dimensions. The first unit, Conv3D, has 128 filters with an input shape of (75,46,140). The second unit of Conv3D has 256 filters, and the last unit of Conv3D has 75 filters. The Conv3D extracts the spatiotemporal characteristics from the given input images. Each image is of input shape 46 × 140. Nonlinear activation function ReLU improves the model’s capacity to learn more complex data. The vanishing gradient problem is resolved with ReLU setting the negative values to zero. The max pooling layer in each unit extracts the most prominent features in the feature map. Reducing the dimensions of the feature map, in turn, reduces the computational complexity of the model. A time-distributed wrapper is applied to each temporal slice of the 3D input to preserve the temporal relationships within the data. The output of the previous 3D convolution and pooling layers is converted into the vector by the time-distributed wrapper and given as an input to the next layer, which is the LSTM layer. In models that deal with processing a sequence of words, a distributed layer is significant prior to a bidirectional LSTM layer. The time-distributed wrapper applies to the operations independently to each timestep of the input sequence. The sequential structure of data are maintained while working with the temporal dimension of the data. Bidirectional LSTMs process the sequential data promptly. The distributed layer applied to the flattened layer before LSTM preserves the data’s sequential structure. The bidirectional LSTM layer has 128 filters and an orthogonal initializer. The dropout layers with a probability of 0.1 added between the bidirectional LSTM layers optimize the number of neurons. The last layer is the dense layer with Softmax as an activation function.

**Figure 3:**
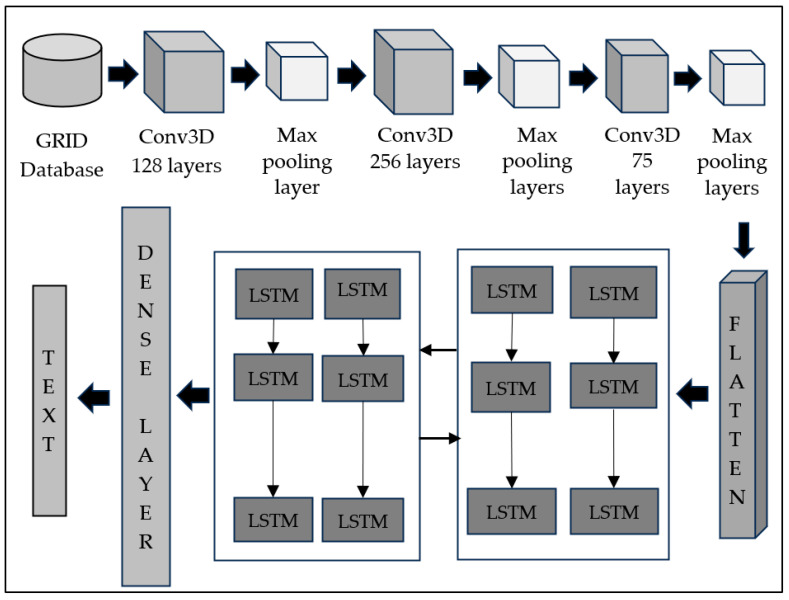
Architecture of proposed speech decoder model in the learning assistive tool.

The decoder predicts the words in the given videos. Connectionist Temporal Classification loss in machine learning allows models to learn the pattern sequence in the data. The CTC loss function is the most suitable in this assistive tool with a deep learning model where the input data have many frames, and there is no exact output corresponding to each frame. Many frames in the input video correspond to one word, and CTC aligns the output word with many frames. Hence, the CTC loss function finds application in speech recognition [27], gesture recognition [28], text recognition [29], and lip reading [30]. The network model is trained with a CTC loss function to give fewer false positives and improved performance. The batch length is computed from the ground truth labels for the batch. The length of the output sequence is the number of labels per sample. CTC loss enables the model to recognize sequences without the need for precise alignment of input frames to output labels, as it computes the probability over all possible alignments for each sample in the batch. In a nutshell, CTC loss is a robust sequence learning tool that utilizes dynamic programming for sequence mapping [31]. Figure 3 shows the architecture of the proposed speech decoder module.

### 3.4. Morse Code Decoder Model

In a deafblind person, communication is haptic. Morse code is one of the haptic methods, and deafblind students understand the Morse code with vibrations. Each letter in the Morse code is a combination of dots and dashes [32]. International Morse codes can be inscribed in braille so that the blind-deaf can initially understand the Morse code for each letter. Once the blind-deaf student practices reading the Morse code in braille, the same knowledge enables them to learn the haptic method [33]. A specially designed wearable device can communicate the Morse code to blind-deaf students. In this proposed assistive tool, text from speech is converted to text with a speech decoder module, and the text is now converted to Morse code and communicated to the visual and hearing-impaired through a wearable device. 

**Figure 4:**
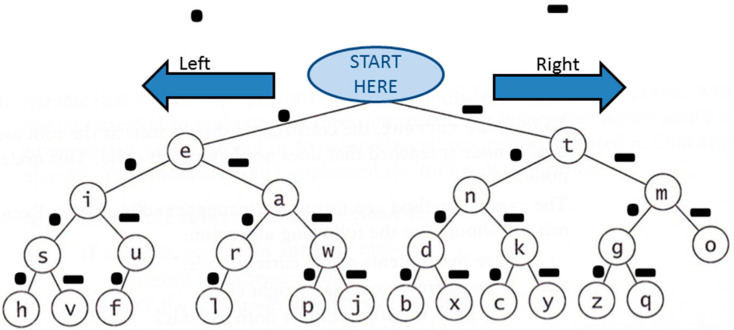
The binary tree representation of the letters and their respective Morse code [34].

The algorithm for conversion of words into Morse code uses the binary tree data structure to convert letters to Morse code. The tree diagram in Figure 4 shows that moving left from any node represents a dot, and moving to the right represents a dash. Visiting the root node and traversing the left branch leads to the letter ‘e’, whereas traversing the right branch leads to the letter ‘t’. The letter ‘f’ can be reached by traversing through the nodes ‘e’, ‘i’, and ‘u’ following a pattern of dot-dot-dash-dot. This tree structure allows accurate conversion of letters to Morse code with inorder, preorder, and postorder traversal algorithms. A similar tree structure for easy conversion also represents the numbers. Figure 4 shows the Morse code representation for each letter and number.

## 4. Experimental Setup and Results

The lip-reading model was trained with a 3D CNN model and bidirectional LSTM network. The 3D CNN model is capable of handling high-volume image sequence data and extracting patterns of lip movements relating to each spoken word. Therefore, 3D CNN was adopted for this study to extract the features of lip movements. The architecture is implemented in a TensorFlow framework with the Keras library [35]. The number of trained parameters is calculated with Equation (1).

(1) $$n_{p}=\left(\left(K_{s}\times \gamma \right)+1\right)\times n_{f}$$

where *K_s_* is the kernel size, γ is the stride value of the kernel, and $n_{f}$ is the number of filters. The first Conv3D layer has 128 filters, the second has 256, and the last has 75. The model has 30 epochs, and the activation function used is ReLU. The model summary shows the output shape and trained parameters for each layer. The trained parameter for the first Conv 3D layer is $\left(\left(3\times 3\times 3\right)+1\right)\times 128=3584$, where the kernel size is (3 × 3), the stride value is 1, and the number of filters is 128. The trained parameters for the second Conv3D layer are (((3×3×3)×128+1)×256)+1=884,992, where the first convolution layer has 128 filters, and the second layer has 256. The number of trained parameters for the last Conv3D layer is (((3×3×3)×256+1)×75)=518,475. The number of parameters $n_{lp}$ is calculated using Equation (2) for bidirectional LSTM.

(2) $$n_{lp}=2\times 4\times (n+m+1)\times m\;\;\mathrm{where}\;n=6375\;\mathrm{and}\;m=128\;\mathrm{units}$$

**Figure 5:**
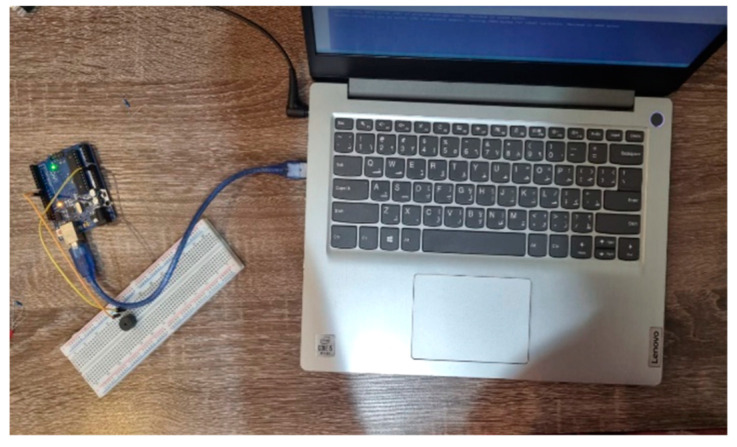
Text to Morse code conversion.

**Figure 6:**
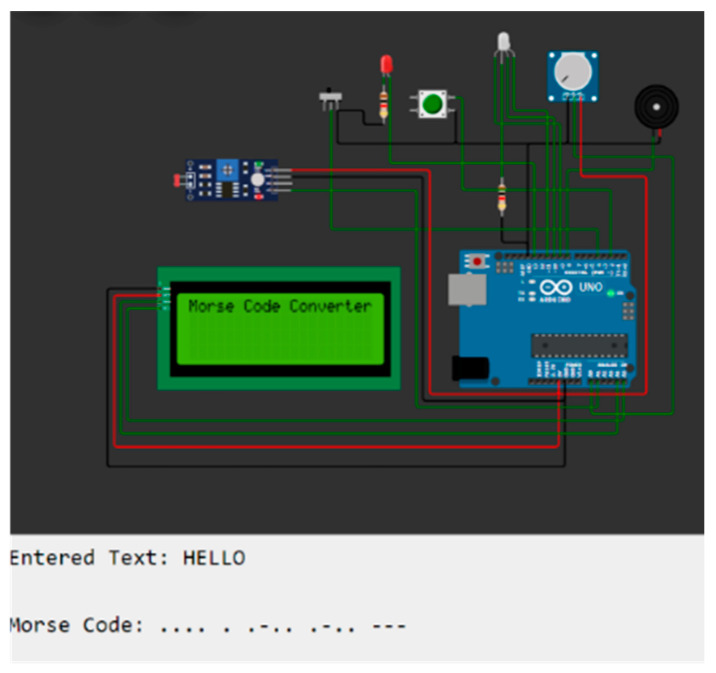
Circuit design to convert words in the text to Morse code.

**Figure 7:**
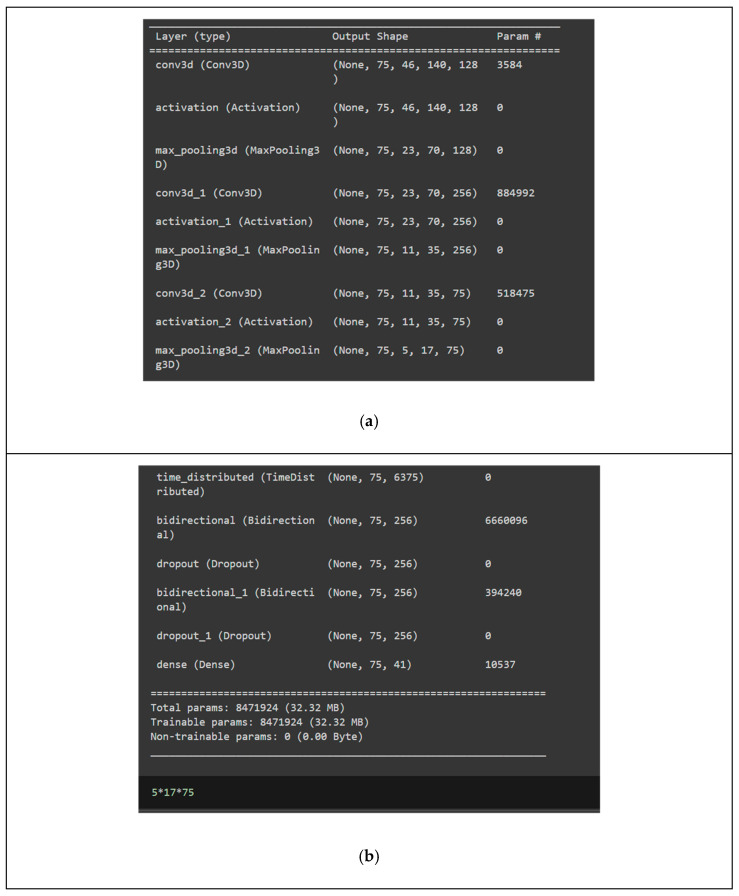
Model summary of proposed (**a**) 3D CNN; (**b**) bidirectional LSTM model.

The input shape is 6375 × 1, the number of units in bidirectional LSTM is 128, and the number of parameters is 6,660,096. In the second LSTM layer, the parameter value of n is 256, m is 128 units, and the $n_{lp}$ is 394,240. In the dense layer, the number of parameters is 10537, and the total number of parameters trained is 8,471,924. Figure 5 shows the image of devices converting text to Morse code. Figure 6 shows the circuit design of the module for the conversion of the recognized text to Morse code. Figure 7 shows the model summary of bidirectional LSTM after 3D CNN.

The GRID Corpus dataset was used to train and test the proposed 3D CNN and bidirectional LSTM model to convert the speech in the videos to the text format. The test dataset includes 1000 videos from the dataset to predict model performance. The ground truth label is prepared, the result is a list of strings, where each string represents a sentence. The input data dimension is converted to match with the model input shape. The predicted output is decoded with CTC with greedy parameter set to “True” to specify the path used is the best one. CTC decoding finds its application in problems where the alignment between the input and output sequence is unknown. The predicted text is compared to the actual text for performance evaluation. It performs a sentence level evaluation.

## 5. Discussion

Word Error Rate (WER) is the standard metric to evaluate the performance of the automated lip recognition system. The word error rate is the ratio of errors that includes the sum of $W_{s}$ number of substitutions, $W_{i\;}$ count of insertions, and $W_{d\;}$ count of deletions to a total number of words $W_{c}$ [36].

(3) $$WER=\frac{\left(W_{s}+W_{i}+W_{d}\right)}{W_{c\;}}$$

Levenshtein distance is the difference between the actual text spoken in the video and the text predicted by the model. It is a similarity measure between two strings, evaluating the count of insertions, deletions, and substitutions to make the strings similar. This method uses the real text and the predicted text as its parameters. The word error rate can be calculated from the Levenshtein distance.

(4) $$WER=\frac{1}{N_{ref}^{*}}\sum_{k=1}^{K}\min d_{L}(r_{k},h_{k})$$

where $N_{ref}^{*}$ is the total number of words in the reference sentence, $r_{k}$ is the reference sentence, $h_{k}$ is the hypothesis sentence, and K is the total number of sentences in the text [37]. True positives, false positives, and false negatives are the metrics for evaluation of the prediction model. TP is incremented when the word predicted is the same as the word in the text; FP is incremented when the word predicted is different from the word in the actual text; and if the word in the actual text is missing in the predicted text, then it is counted as FN. The equations for accuracy, precision, recall rate, and f1-score for all text recognition are given with Equations (5)–(8).

(5) accuracy=1−WER

(6) $$precision=\frac{TP}{TP+FP}$$

(7) $$recall\_rate=\frac{TP}{TP+FN}$$

(8) $$f1-score=\frac{2\times precision\times recall\_rate}{precision+recall\_rate}$$

**Figure 8:**
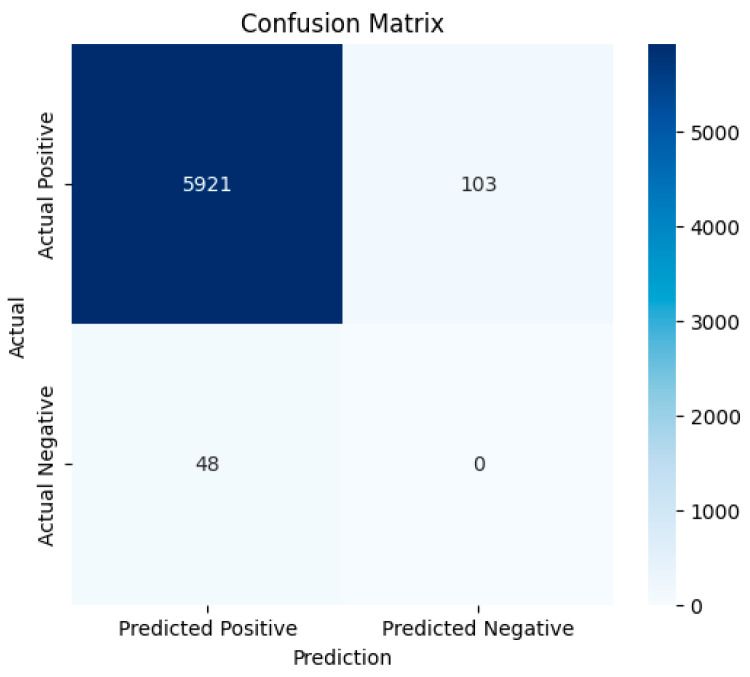
Confusion matrix for the proposed model.

Table 1 compares the LipNet model with the proposed system. The LipNet model shows WER of 4.8% where the proposed model shows the WER of 2%. The confusion matrix displayed in Figure 8 shows that the number of true positives is higher than the false positives and false negatives, indicating that the predicted text is highly similar to the content spoken in the video. The precision of the model is 99%, which indicates that the number of false positives is negligible and that the model has accurately detected most of the words in the dataset. The false negatives are the count of words available in the actual text but missing in the predicted text. The false negative count is double the false positive count; hence, the recall rate is reduced to 98%. The negligible count of false positives and negatives contribute to the high precision, recall rate, and f1-score metrics. The confusion matrix indicates that the model’s performance is excellent for the extensive test data considered for this study. The confusion matrix clearly indicates that 6072 sentences were evaluated in the experimental study. The confusion matrix shows that the number of true positives TP is 5921, false positives FP is 48, and false negatives FN is 103. The recall rate is the ratio of TP to the number of TP and FN. The high number of true positives leads to a higher recall rate than the existing state-of-the-art models.

Any classification or prediction problems in the medical diagnosis domain demand a high recall rate, as missing a positive case may be a fatal error. The model has high precision compared to the recall rate, indicating fewer false positive errors but more false negatives. F1-score manages the trade-off between the recall rate and precision. The F1-score is the harmonic mean value of recall rate and precision. A higher F1 score indicates a reasonable recall rate and precision balance. The accuracy is 98%, the ratio of true positives to the word count. The average WER is 2%, indicating that the model performs exceptionally well compared to the state-of-the-art methodology. Table 2 shows the state-of-the-art methodologies for lip recognition. The proposed model shows high performance compared to the other state-of-the-art methodologies, which have acquired high accuracy at the sentence level.

The research study by Jeong [42] adopts a pre-trained model and considers the speakers at both vision and language levels. This study integrates prompt tuning and the LoRA approach for sentence-level lip reading in English with a word error rate of 40.9% by utilizing adaptations on both vision and language levels for the target speaker. The study proposes an innovative model built with 3D CNNs LSTM networks to tackle word recognition from lip movements. The dataset for the study is MobLip, which has speech patterns under different environmental conditions. The model shows an accuracy of 87.5%, leveraging diverse speeches and lighting conditions [43]. The proposed lip recognition model has acquired an accuracy of 98% at the word level. The proposed model uses data prefetching techniques to boost processor performance and improve resource utilization during training. The data shuffling restrains the model from learning spurious patterns from the train data, thereby preventing the model from predicting the words based on frequently occurring sentence patterns. The vanishing gradient problem is also handled in the model architecture by the ReLU activation function. The time distributed wrapper layer prior to LSTM preserves the sequential structure of the data and is one of the significant factors in improving the model’s accuracy.

## 6. Conclusions

The proposed assistive tool enables visual and hearing-impaired students to learn directly from the teacher in a classroom rather than facing the overwhelming tactile teaching method. Teaching abstract concepts in mathematics, science, and technology using tactile methods is challenging. A significant drawback in imparting quality education is the lack of experienced and skilled teachers to educate visually and hearing-impaired students. This assistive tool enables the visually and hearing impaired to learn from experienced and highly qualified teachers like any other students in a reputable institution with the appropriate facilities. This assistive tool is a breakthrough in the field of education concerning visually and hearing-impaired children in gaining knowledge and performing at par with children who are not disabled.

## Figures and Tables

**Table 1:** Performance evaluation of the proposed model.

Study	Methodology	Recall_Rate	Precision	F1-Score	Accuracy
Assael, Y.M [15]	LipNet (GRU)	-	-	-	95.2%
Lu, Y et al. [16]	VGG19 + LSTM	-	-	-	88.2%
SriKanth, G.N et al. [18]		-	-	-	91%
Yafeng, Y et al. [38]		-	-	-	91.58%
Aripin et al. [39]	LRCN + 3D Conv	-	-	-	95.4%
Zhou, H [40]	LPRNet	-	-	-	97.9%
Proposed Model		98%	99%	99%	98%

**Table 2:** Comparative study with state-of-the-art methodologies.

Study	Year	Dataset	Methodology	Performance
Kim, M. et al. [41]	2024	GRID	3D CNN and Transformer	WER: 3.13%
Yeo, J.H. et al. [42]	2024	VoxLRS-SA	LLM	WER: 40.9%
Exarchos, T. et al. [43]	2024	MobLip	3D CNN and LSTM	WER: 12.5%
Proposed methodology	2024	GRID	3D CNN and Bidirectional LSTM	2% WER

## Data Availability

Data openly available in a public repository. The data that support the findings of this study are openly available in The GRID audio-visual sentence corpus at https://spandh.dcs.shef.ac.uk/gridcorpus/ (accessed on 5 January 2024).
